# Awake rabbit model of ischemic spinal cord injury with delayed paraplegia: The role of ambient temperature

**DOI:** 10.1002/ame2.12346

**Published:** 2023-09-11

**Authors:** Wang Yang, Qian‐qian Wu, Lu Yang, Yu‐jie Chen, Ren‐qing Jiang, Ling Zou, Qing‐shan Liu, Guang‐you Shi, Jiang Cao, Xiao‐chao Yang, Jian Sun

**Affiliations:** ^1^ School of Biomedical Engineering and Medical Imaging Army Medical University Chongqing China; ^2^ Army Health Service Training Base Army Medical University Chongqing China; ^3^ Department of Neurosurgery, Southwest Hospital Army Medical University Chongqing China

**Keywords:** ambient temperature, delayed paraplegia, rabbit model, spinal cord injury, spinal cord ischemia

## Abstract

**Background:**

Paraplegia after spinal cord ischemia is a devastating condition in the clinic. Here, we develop an awake rabbit model of spinal cord ischemia with delayed paraplegia and explore the influence of ambient temperature on the outcomes after injury.

**Methods:**

A total of 47 male rabbits were involved in the present study. Transient spinal cord ischemia was induced by occluding the infrarenal abdominal aorta of awake rabbits at different ambient temperatures. To find the optimal conditions for developing delayed paraplegia, hindlimb motor function after ischemia was evaluated between experiments.

**Results:**

The onset and magnitude of ischemic injury varied with the ambient temperature maintained during the peri‐ischemia period. More serious spinal cord injury occurred when ischemia was induced at higher temperatures. At 18°C, 25‐minute ischemia resulted in 74% of rabbits developing delayed paraplegia. At a temperature of 28°C or higher, most of the animals developed acute paraplegia immediately. While at 13°C, rabbits usually regained normal motor function without paraplegia.

**Conclusion:**

This awake rabbit model is highly reproducible and will be helpful in future studies of delayed paraplegia after spinal cord ischemia. The ambient temperature must be considered while using this model during investigation of therapeutic interventions.

## Graphical abstract

The magnitude of ischemic spinal cord injury varied with the ambient temperature maintained during ischemia. More serious injury occurred when ischemia was induced at higher temperatures. At 18°C, 25‐minute ischemia resulted in most rabbits developing delayed paraplegia.

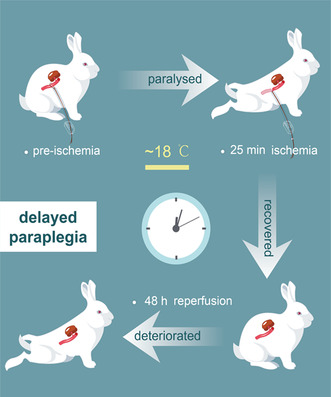

## 1 INTRODUCTION

Ischemic spinal cord injury (ISCI) with paraplegia is a devastating complication of thoracoabdominal aortic aneurysm repair. Although much progress in perioperative prevention and management has been made, ISCI is still considered to be the Achilles' heel of aortic repair and the incidence rates remain at approximately 4%~10%.^1^, ^2^, ^3^ Paraplegia after ISCI can be both acute and delayed. In some cases, acute‐onset paraplegia occurs immediately after patients emerge from anesthesia after the aortic surgery, and is nearly irreversible; by contrast, delayed‐onset paraplegia typically develops several days later, providing a broad therapeutic window for prevention and intervention.^4^ However to date, there is no way of knowing whether delayed paraplegia will occur in those patients who initially appear to have normal neurological function after the surgery. Besides, available information about the pathological mechanism responsible for delayed paraplegia is limited, which in part, stems from the lack of a highly reproducible animal model.

Although various experimental models of ISCI have been developed, none is ideal for exploring the pathological mechanisms of delayed paraplegia, because most of them present either few symptoms or acute paraplegia without delay.^5^, ^6^, ^7^, ^8^ In addition, spinal cord ischemia in these models was conducted during anesthesia, which did not exclude possible neuroprotective effects of anesthetics.^9^ In the 1980s, delayed paraplegia was noticed in an awake rabbit model of spinal cord ischemia. Zivin et al. first developed the model and reported about 40% of the rabbits showed evolving spinal cord deficits after 25~30 min occlusion of the infrarenal abdominal aorta.^10^ Using the same method, Jacobs et al. found 70% of the rabbits developed delayed paraplegia after 25‐minute ischemia, while 20 min was not enough to achieve it.^11^ Hollier et al. also reported that most animals developed delayed paraplegia but in their study the optimal occlusion time was 20~21 min; 25~30 min of occlusion usually resulted in acute paraplegia.^12^ One year later, in another study conducted by the same laboratory, only 30% of the rabbits in the control group developed delayed paraplegia after 20~21 min ischemia.^9^ Using the same method to induce ISCI, significant discrepancies in outcome were seen among these studies, particularly regarding the optimal occlusion time for developing delayed paraplegia and the percentage occurrence. These discrepancies could in part be attributed to individual differences, but other factors during the perioperative period might also have played a role in determining the outcome.

In the present study, we have established a highly reproducible awake rabbit model of ISCI with delayed paraplegia. In addition to occlusion time, we showed that the ambient temperature, which can easily be ignored during the construction of animal models, plays a critical role in the onset and magnitude of paraplegia, which might shed some light on the discrepancies in the outcome of the studies mentioned above. When spinal cord ischemia was induced under different ambient temperatures, the onset and magnitude of paraplegia varied. Generally, the spinal cord injury became more serious with increasing ambient temperature. Furthermore, by changing the ambient temperature from 13°C to 33°C, we have discovered the optimal conditions for facilitating the occurrence of delayed paraplegia.

## 2 METHODS

### 2.1 Animals and surgical preparation

Forty‐seven male New Zealand White rabbits (body weight, 1.9–2.7 kg) obtained from the Laboratory Animals Center of the Army Medical University were involved in these experiments.

**FIGURE 1:**
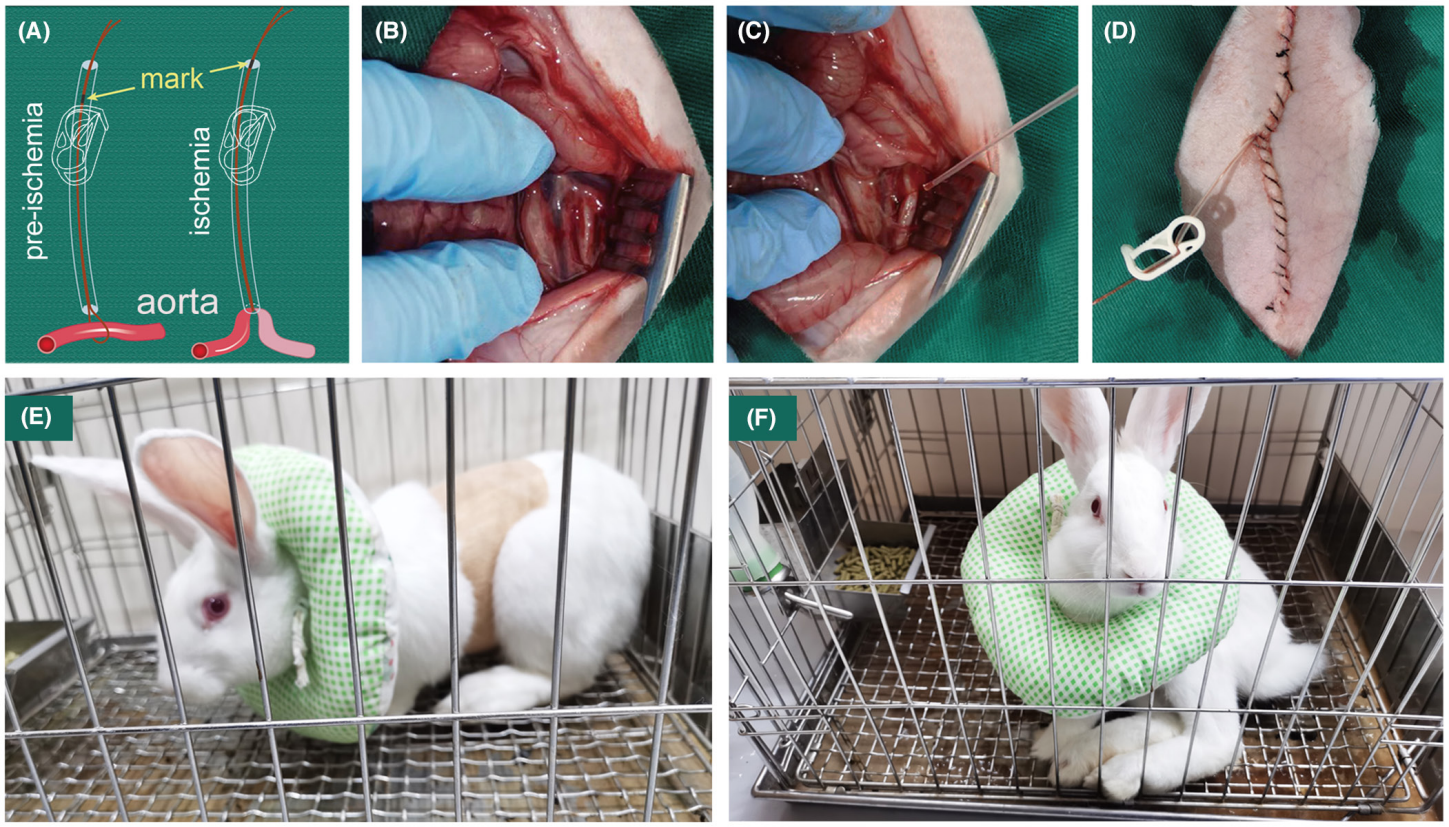
(A), Schematic illustration of inducing ischemia with the snare ligature: the aorta is occluded by pulling tight the custom‐made snare ligature with permanent waterproof marks. (B–D) provides a brief description of the surgery: the infrarenal abdominal aorta is isolated; the snare ligature is placed around the aorta; the incision is closed and the external side of the snare is clamped. (E), Pre‐ischemia: the animal awakens from the surgery with normal hindlimb motor function. (F), During ischemia: animal presents complete paralysis.

After an overnight fast with free access to water, animals were anesthetized by intravenous administration of 30 mg/kg sodium pentobarbital. In the supine position, a midline, vertical abdominal incision was performed and the aorta was isolated at the level of the renal artery. A custom‐made snare ligature occluding device was implanted and loosely encircled the aorta as shown in Figure 1. In detail, a monofilament nylon line (diameter 0.2 mm) was placed around the aorta just distal to the left renal artery. The ends of the monofilament were threaded through a PE 90 polyethylene catheter (length, 12~14 cm; internal diameter, 0.86 mm; outer diameter, 1.32 mm), forming a snare ligature. The internal side of the catheter was placed near the aorta with the other side remained outside the abdomen clamped by a medical Robert clamp. The incision was then closed, with the external part of the snare remaining accessible. The Robert clamp here had the dual function of preventing the external part of the snare from moving into the abdomen through the incision site as well as a relative slip between the monofilament and the catheter. After the operation, a dose of gentamicin (40 000 IU) was injected intramuscularly as a prophylactic antibiotic. Each rabbit was then fitted with an Elizabethan collar and its abdomen was protected by self‐adhesive elastic bandages. The animals were then randomized into different groups and returned to cages under five controlled ambient temperatures (13°C, 18°C, 23°C, 28°C, 33°C, with ±0.5°C variation).

### 2.2 Induction of spinal cord ischemia

Spinal cord ischemia was induced by tightening and clamping the snare ligature. Briefly, about 20 h after surgery, all rabbits had completely recovered from anesthesia. The Robert clamp was then opened and the snare ligature was gently pulled until permanent waterproof marks on the monofilament, made before the implantation of the snare to prevent rupture of the aorta caused by excessive pulling, reached the end of the catheter. When some resistance was felt, the ligature and the catheter were clamped with the Robert clamp again, thus occluding the infrarenal abdominal aorta. Adequate ischemia could be confirmed by observation of complete hindlimb paralysis. After occlusion for 25 min, the snare ligature was released and removed from the abdomen through the incision site. Restoration of blood flow was confirmed by direct touch of the femoral artery. After reperfusion, animals were housed in a feeding room with a standard pellet diet. Postoperative observations were conducted, and the Crede maneuver was performed if necessary.

### 2.3 Neurological evaluation

At 1, 4, 12, 24, and 48 h during the reperfusion period, the hindlimb motor function was evaluated by an observer who was blinded to the group allocation, using the following modified Tarlov score^11^: 0, complete paralysis; 1, severe paresis, minimal functional movement; 2, active movement but no hopping; 3, hopping with obvious ataxia and/or paresis; 4, hopping with minimal ataxia and/or paresis (or less sensitive to noxious stimuli); 5, hopping normally.

### 2.4 Histological analysis

After finishing the last hindlimb motor function evaluation, 5 representative animals of each ISCI type were euthanized by intravenous injection of 100 mg/kg sodium pentobarbital. The lumbar spinal cords (L5 level) were rapidly removed and fixed with 4% paraformaldehyde in 0.1 M phosphate buffer at 4°C for about 1 week. The specimens were then embedded in paraffin and cut into coronal sections of 5 μm thickness for hematoxylin–eosin (HE) staining and terminal deoxynucleotidyl transferase‐mediated dUTP nick‐end labeling (TUNEL) assay. The images were acquired on an Olympus Slide VS200 whole slide scanner.

For quantitative analysis, the number of normal neurons and TUNEL‐positive cells in the anterior spinal cord (anterior to a line drawn through the central canal perpendicular to the vertebral axis) were counted by an observer who was blinded to the group allocation. Then, based on the number of TUNEL‐positive cells, a TUNEL‐positive score was allocated as a measure of the extent of apoptosis: 1, no or little staining (number of TUNEL‐positive cells, 0~5); 2, slightly stained (number of TUNEL‐positive cells, 5~25); 3, moderately stained (number of TUNEL‐positive cells, 25~125); 4, densely stained (number of TUNEL‐positive cells, above 125).

### 2.5 Statistical analysis

Statistical analysis was performed with Prism 9. The number of normal neurons and the TUNEL‐positive score were analyzed using a nonparametric method (Kruskal–Wallis test) followed by the Mann–Whitney *U* test. *p* < 0.05 was considered statistically significant.

## 3 RESULTS

### 3.1 ISCI types and hindlimb motor function

All rabbits became completely paraplegic within 2 min of tightening the ligature. During the 48‐h reperfusion period, three types of ISCI occurred:

Type 1, acute‐onset paraplegia. After reperfusion, the rabbit remained in severe paralysis and was permanently unable to hop (motor score ≤2).

Type 2, delayed‐onset paraplegia. After reperfusion, the rabbit regained hopping function initially (motor score ≥3), but then became paraplegic, gradually losing the ability to hop.

Type 3, no paraplegia. After reperfusion, the rabbit regained hopping function and remained able to hop during the whole reperfusion period.

**FIGURE 2:**
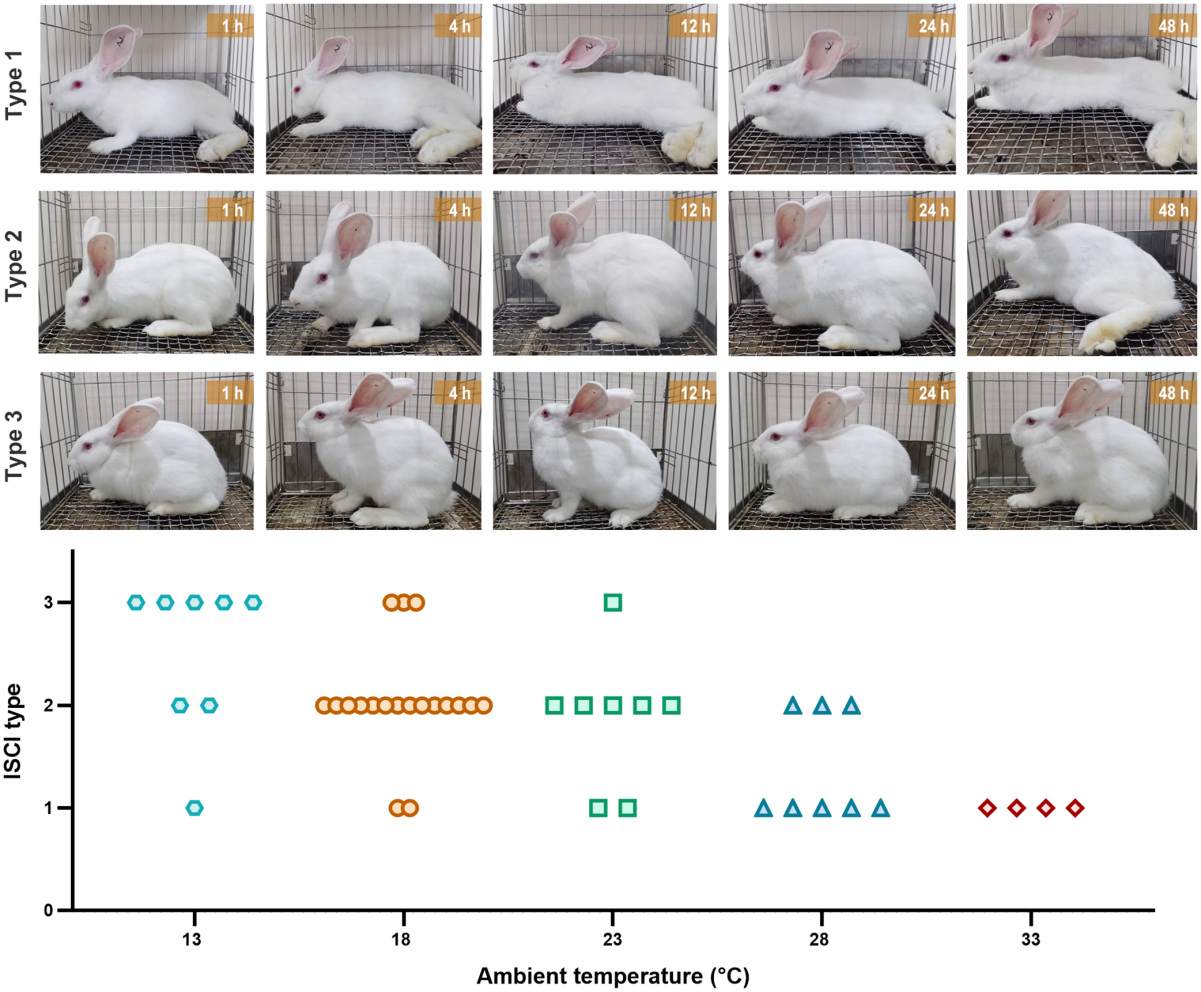
Representative photos of three Ischemic spinal cord injury types and their distributions in different groups during the 48‐h reperfusion period.

The numbers of each of these types in the different temperature groups are shown in Figure 2.

**13°C**. Most rabbits (*n* = 7/8) regained hopping function within 1 h of reperfusion and only 2 of them became paraplegic at 48 h. Acute paraplegia occurred in 1 rabbit occurred in this group.

**18°C**. Most rabbits (*n* = 14/19) developed delayed paraplegia. At 1 h, 14 rabbits had regained hopping function but 11 of them hopped with obvious ataxia. At 4 h, 17 rabbits could hop; 14 of them hopped normally or with minimal ataxia, while the other 3 hopped with obvious ataxia. The rabbits usually retained motor function for longer than 12 h. Subsequently, a secondary decline in motor function occurred in 74% (*n* = 14/19) of the rabbits, 6 and 8 of them developed paraplegia at 24 h and 48 h respectively. Three rabbits retained hopping function until they were euthanized while 2 rabbits suffered acute paraplegia in this group.

**23°C**. Many rabbits (*n* = 5/8) in this group developed delayed paraplegia. The motor function showed a progressive decline, similar to that of the 18°C group, but a slower recovery and severer impairment were noticed. Specifically, at 1 h, only 3 rabbits had regained hopping function. At 4 h, 3 more rabbits regained hopping function but hopped with obvious ataxia. By 12 h after reperfusion, 1 rabbit had lost the ability to hop, while by 24 h, another 4 rabbits became paraplegic, and 1 rabbit still hopped normally. However, over the next 24 h, the rabbit that hopped normally showed some deterioration and hopped with ataxia. By 48 h after reperfusion 2 rabbits developed acute paraplegia and could not hop at all.

**28°C**. Many rabbits (*n* = 5/8) developed acute paraplegia. Much lower motor scores were obtained after reperfusion. Regardless of the severity of the impairment, none of the rabbits regained normal motor function during the observation period. Only 3 of 8 rabbits recovered hopping function initially, and 1 of them became paraplegic within 12 h while the other 2 became paraplegic within 48 h.

**33°C**. In this group, all the rabbits (*n* = 4) developed acute paraplegia immediately and none recovered.

**FIGURE 3:**
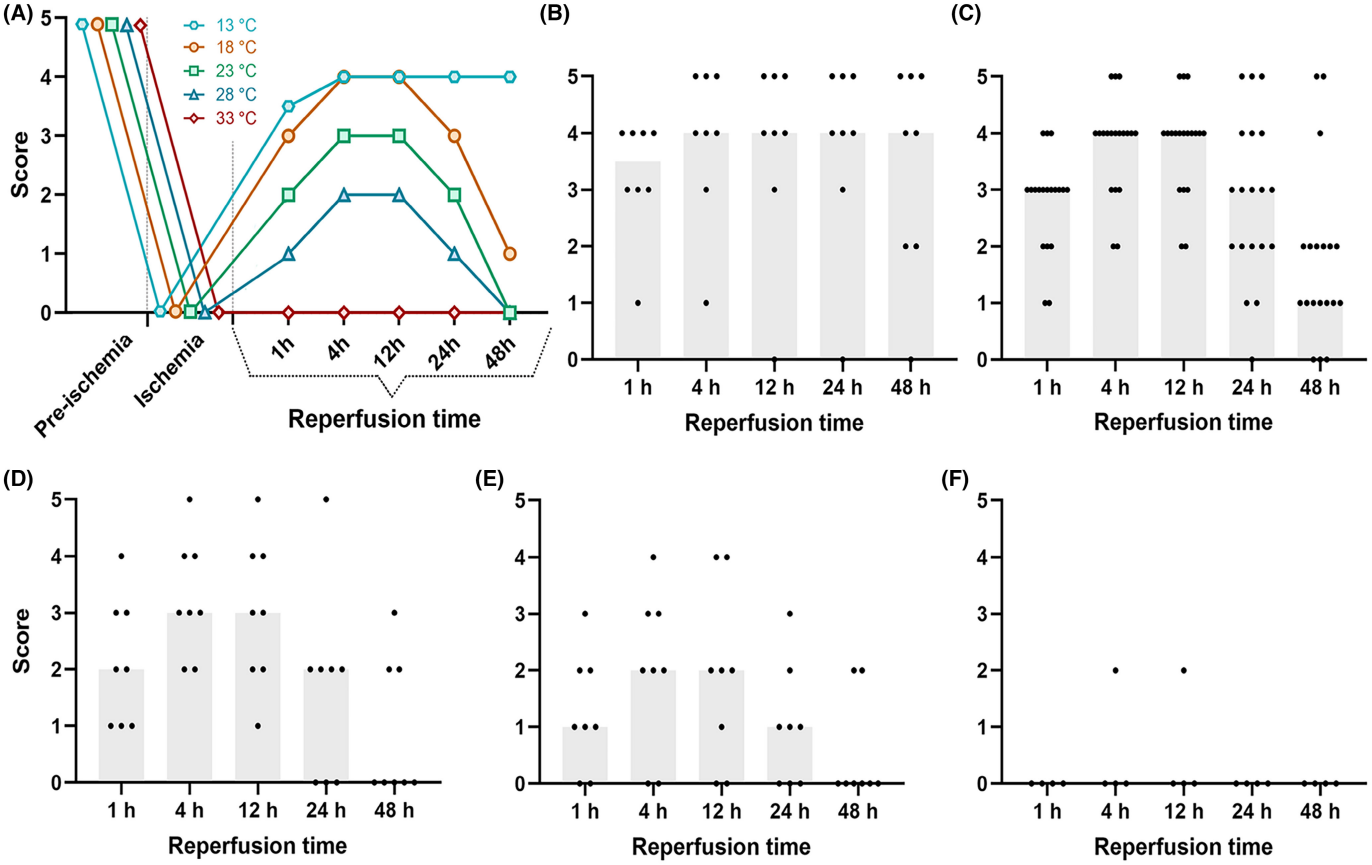
(A), Hindlimb motor score evaluated during the ischemic‐reperfusion period in the different groups and at each temperature. (B), Scores at 13°C (*n* = 8). (C), Scores at 18°C (*n* = 19). (D) 23°C (*n* = 8). (E) 28°C (*n* = 8). (F) 33°C (*n* = 4). The points are median neurological scores.

Generally, with increasing temperature, the motor score during the reperfusion period became lower, as shown in Figure 3 (see Table S1 for details).

### 3.2 Histopathological outcome

**FIGURE 4:**
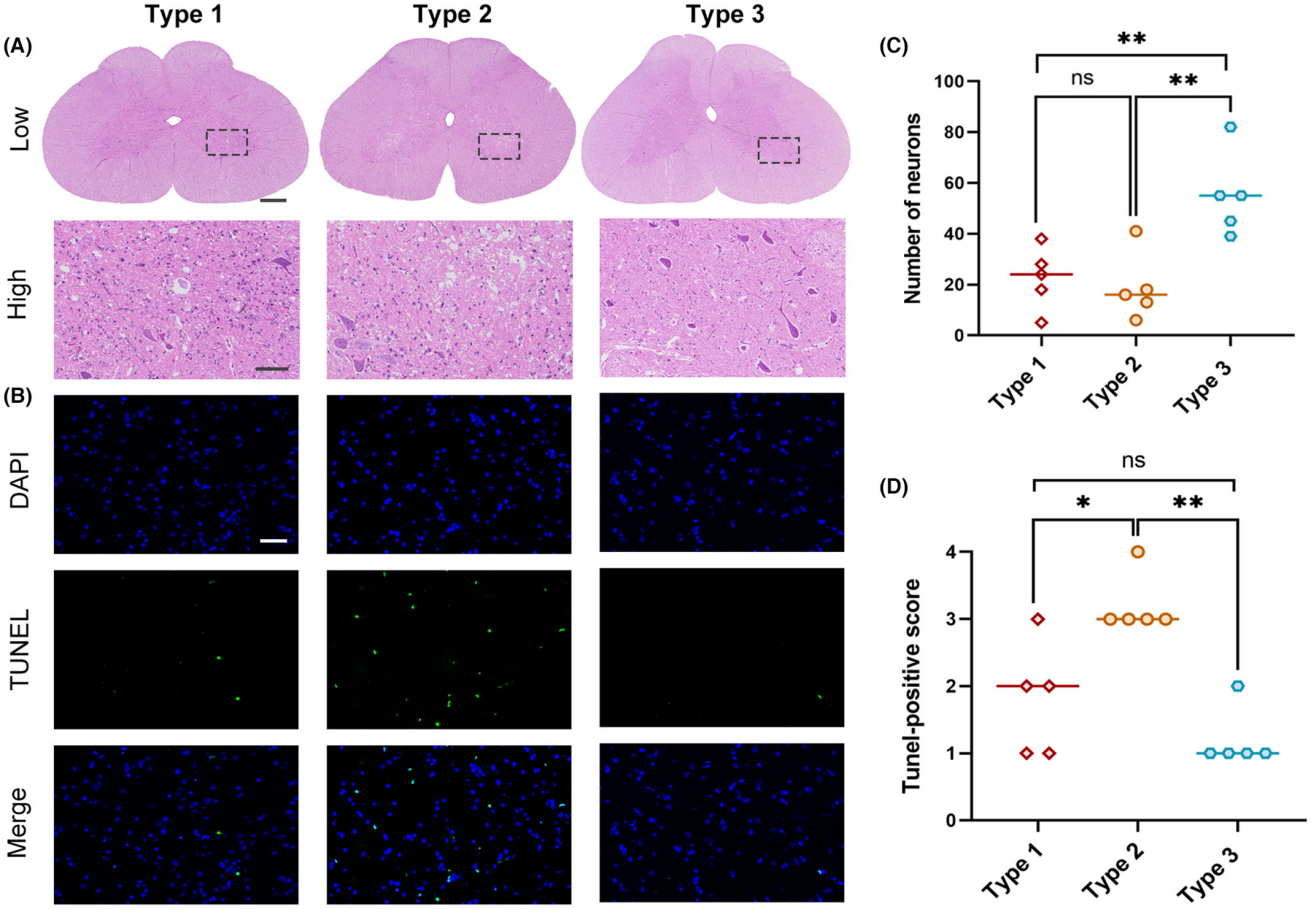
Histopathological results in different Ischemic spinal cord injury (ISCI) types (*n* = 5). Representative images of the spinal cord at 48 h stained by HE (A) and TUNEL (B). HE, Scale bars = 500 μm for low resolution images and 100 μm for high resolution images; TUNEL, 50 μm. (C) and (D), The number of normal neurons (C) and TUNEL‐positive scores (D) in the spinal cord of different ISCI types. Data are presented as median values. *p < 0.05 and **p < 0.01.

The intraspinal pathological outcome after 25‐minute occlusion of the infrarenal aorta was qualitatively and quantitatively analyzed by histological staining methods (5 rabbits of each type were selected). The results of HE and TUNEL staining showed big differences among the three types, as shown in Figure 4 (see Table S2 for details). There were significantly fewer normal motor neurons in the anterior spinal cord of type 1 and 2 rabbits than that of type 3. However, no significant difference was found between the type 1 and 2 rabbits by 48 h after reperfusion. Few TUNEL‐positive cells were noticed in the spinal cords of type 1 and 3 rabbits, while obvious TUNEL‐positive cells were noticed in the type 2 rabbits after 48 h of reperfusion, indicating that apoptosis might play a role in the onset of delayed paraplegia.

## 4 DISCUSSION

Delayed paraplegia after spinal cord ischemia has rarely been studied due to the lack of a suitable animal model. In 2010, a mouse model of delayed paraplegia was developed by downregulating the core temperature, which might indicate some similarity in mechanisms with this awake rabbit model.^13^ Although the mouse model offers the advantage of using gene knock‐in and knockout techniques to explore the pathology of ISCI, occlusion of the thoracic aorta causes ischemic damage not only to the spinal cord but also to the anoxia‐sensitive kidneys. The induction of ISCI in rabbits by ligation of the infrarenal abdominal aorta therefore offers a better model. Moreover, the awake rabbit model possesses a twofold advantage for studying spinal cord ischemia. First, it removes the influence of anesthetic agents, which might have some protective effects on neurons; second, it offers a unique opportunity to observe in real time the changes in hindlimb motor function over the entire ischemia–reperfusion period. Although the first awake rabbit model of ISCI was reported several decades ago, it has not been used until recently due to weak reproducibility and stability. Specifically, variability of outcomes among different laboratories has been noted.^10^, ^11^, ^12^ These variations could partly stem from individual differences in ischemic injury severity, but other factors during the ischemia–reperfusion period might also play an important role in determining the outcome.

In the present study, we found that ambient temperature during ischemia is important for development of delayed paraplegia in the rabbit; motor function was closely related to the ambient temperature. When the ischemia was conducted at a temperature as low as 13°C, most rabbits recovered normal motor function quickly and developed no paraplegia. By contrast, at relatively high temperatures, rabbits recovered motor function slowly (at 23°C), while at the highest temperatures most developed acute paraplegia immediately (at 28°C and 33°C). The percentage occurrence of delayed paraplegia after spinal cord ischemia conducted at 13°C, 18°C, 23°C, 28°C, and 33°C was 25%, 74%, 62.5%, 37.5%, and 0% respectively. For patients, recommended ambient temperatures during surgery range from 17°C to 27°C according to the type of surgery,^14^, ^15^ while the desirable temperature for rabbits is between 13°C and 20°C.^16^ Therefore, there are likely to have been inconsistencies in ambient temperatures between some experimental studies and different laboratories, thus leading to the variability of outcomes in previous studies. How does ambient temperature influence the outcome of ISCI in this awake model? We hypothesize that the metabolic and thermoregulatory responses to temperature change might answer this question. At different temperatures, adaptive changes of physiological metabolism and thermoregulation can occur in rabbits during awake states^17^, ^18^, ^19^; these include respiration rates, metabolic rates, body temperature, and other physiological parameters, all of which might directly or indirectly influence the severity of injury after central nervous system ischemia.^20^, ^21^, ^22^ As the environmental temperature decreases, the metabolic rate will increase to maintain the balance of body temperature. But under the condition of ischemia, the oxygen and nutrition supplied to the corresponding tissues are disrupted, leading to metabolic disorders and an imbalance of heat production. As a result, heat exchange between the local ischemic area and the outer environment is going to occur. For instance, without a heat source around the brain the brain temperature will decline spontaneously during ischemia, and even small changes are enough to influence the severity of ischemic damage and the metabolic response to ischemia.^22^ In addition, the extent of the decline in brain temperature seems to be greater as the environmental temperature is lowered.^21^ As the spinal cord shares many similar characteristics with the brain, it is possible that the spinal cord temperature during ischemia varies at different ambient temperatures, which might explain the various outcome obtained in the present study. It is well known that hypothermia has some protective effects on spinal cord injury.^23^ Therefore, in any study of central nervous system ischemia, the temperature should be consistent to exclude the possible interference of the temperature effects. Technically it is difficult for us to monitor the local spinal cord temperature during ischemia induced under conscious states. Although lower rectal temperatures were noticed after 25 min of ischemia compared with pre‐ischemia, there was no significant difference between the groups housed at 13~28°C, but the rectal temperatures of rabbits in the 33°C group were higher than other groups (Figure S1), which was probably due to their being less tolerant of temperatures as high as 33°C.^19^ Nevertheless, the rectal temperature cannot reliably reflect spinal cord temperature during ischemia.^24^ Unlike the rectal temperature, the respiration rate correlated well with the ambient temperature (Figure S2), indicating that a thermoregulatory response to different temperatures occurred, as previously reported.^17^, ^18^

In addition to exploring the ambient temperature effects on the outcome of ISCI, we have established an experimental animal model of delayed paraplegia. The most favorable condition for establishing delayed paraplegia is 25 min of ischemia at nearly 18°C, a desirable temperature for rabbits. We also tested 20 min of ischemia at 18°C and 23°C, after which about 37.5% and 62.5% of animals developed delayed paraplegia, respectively (data not shown). Since 23°C exceeds the suitable temperature range for rabbits (13~20°C), we do not recommend it. In another study, a rabbit model of delayed paraplegia was established during anesthesia, with a shorter ischemia time of 15 min,^25^ mainly because the spinal cord temperature was controlled by a heat source during anesthesia. Based on the literature and our study, we conclude that the severity and onset of paraplegia are a function of spinal cord temperature and ischemia time. Therefore, by changing these two parameters, different outcomes can be obtained. With regard to the three types of ISCI in our study, the histopathology was quite different. Our HE and TUNEL staining results showed that motor score correlated well with the onset and magnitude of the injury, providing an easy way to evaluate intraspinal injury by direct observation of hindlimb performance. It is likely that necrosis occurs more in acute paraplegia as a result of long‐term ischemia, while apoptosis plays an important role in the development of delayed paraplegia during the reperfusion period.^25^, ^26^ After reperfusion, normal neurological function remains for longer than 12 h, allowing us a broad therapeutic window for interventions, such as preventing apoptosis.^27^

## 5 CONCLUSION

In conclusion, a highly reproducible animal model of delayed‐onset paraplegia has been established. The ambient temperature, which is a factor that can be easily ignored during the cross‐clamp of the lower abdominal aorta, has to be considered while using this model during the investigation of therapeutic interventions. Although the mechanism underlying the effects of ambient temperature needs to be further elucidated, we believe this awake rabbit model will be helpful in future studies of ISCI with delayed paraplegia.

## AUTHOR CONTRIBUTIONS

Jian Sun, Xiaochao Yang, and Wang Yang conceived and designed the experiments. Wang Yang, Qianqian Wu, Lu Yang, Ling Zou and Jiang Cao conducted all the experiments. Wang Yang, Yujie Chen, Renqing Jiang, Qingshan Liu and Guangyou Shi analyzed the data. Wang Yang and Qianqian Wu prepared the draft manuscript. All authors provided suggestions and contributed to the final manuscript.

## FUNDING INFORMATION

This work was supported by the Science and Technology Research Project (KJQN202212805) of the Chongqing Education Commission and the Special Funding Project (2021XJS08) of Army Medical University.

## CONFLICT OF INTEREST STATEMENT

The authors declare no conflicts of interest.

## ETHICS STATEMENT

All procedures were approved by and performed following the Guidelines for Care and Use of Laboratory Animals of Army Medical University (Approval No. AMUWEC20228022).

## Supporting information


Figure S1.

## Data Availability

Raw data supporting this study are available from the corresponding author upon reasonable request.
