# Supplementary material for: Awake rabbit model of ischemic spinal cord injury with delayed paraplegia: The role of ambient temperature
Source: Animal Model Exp Med. 2023 Sep 11;7(5):732–9. doi: 10.1002/ame2.12346 (PMC11528386; doi:10.1002/ame2.12346)
Supplement: Supplementary file 1 — Figure S1. [file AME2-7-732-s001.docx]

Supplementary Material

Awake rabbit model of ischemic spinal cord injury with delayed paraplegia: a role of the ambient temperature

Wang Yang^1, 2†^, Qian-qian Wu^1†^, Lu Yang^1^, Yu-jie Chen^3^, Ren-qing Jiang^2^, Ling Zou^1^, Qing-shan Liu^1^, Guang-you Shi^1^, Jiang Cao^1^, Xiao-chao Yang^1*^ and Jian Sun^1*^

*** Correspondence:** Jian Sun: sunjian@tmmu.edu.cn; Xiaochao Yang: xcyang@tmmu.edu.cn


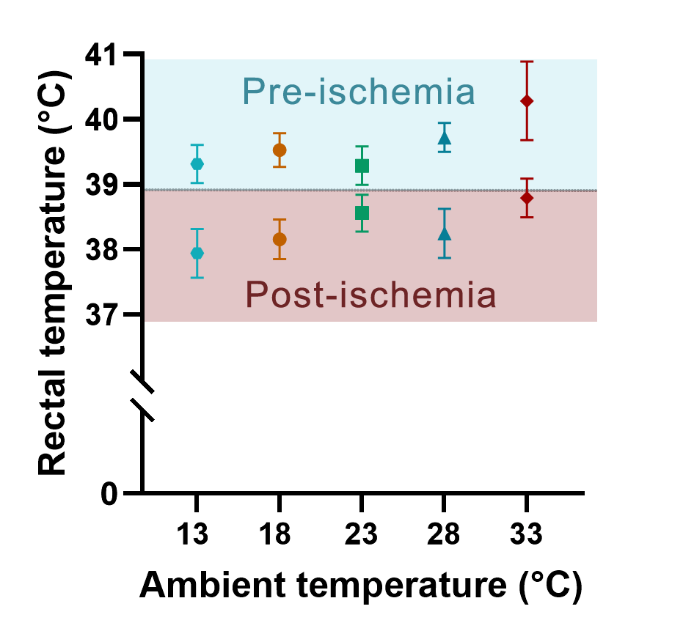


**Figure S1.** The rectal temperature measured at different ambient temperatures.


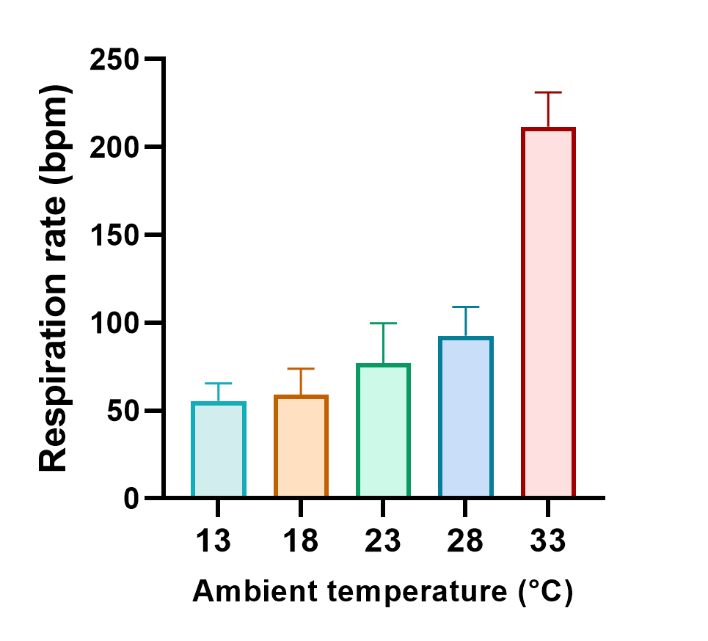


**Figure S2.** The respiration rates measured at different ambient temperatures.

**Table S1.** Neurological function after 25-minute ischemia

| Group | Motor score during reperfusion period | | | | | ISCI type |
| --- | --- | --- | --- | --- | --- | --- |
|  | 1h | 4h | 12h | 24h | 48h |  |
| 13 °C (n=8) | 4 | 4 | 4 | 4 | 4 | 3 |
|  | 4 | 4 | 4 | 4 | 2 | 2 |
|  | 4 | 5 | 5 | 5 | 5 | 3 |
|  | 3 | 5 | 5 | 5 | 5 | 3 |
|  | 3 | 3 | 3 | 3 | 2 | 2 |
|  | 3 | 4 | 4 | 4 | 4 | 3 |
|  | 3 | 4 | 4 | 4 | 4 | 3 |
|  | 1 | 1 | 0 | 0 | 0 | 1 |
| 18 °C (n=19) | 3 | 4 | 4 | 3 | 1 | 2 |
|  | 3 | 3 | 3 | 0 | 0 | 2 |
|  | 3 | 4 | 4 | 4 | 1 | 2 |
|  | 3 | 4 | 4 | 3 | 1 | 2 |
|  | 3 | 4 | 4 | 3 | 1 | 2 |
|  | 3 | 4 | 4 | 4 | 4 | 3 |
|  | 2 | 4 | 4 | 3 | 2 | 2 |
|  | 3 | 4 | 4 | 2 | 2 | 2 |
|  | 4 | 4 | 4 | 1 | 1 | 2 |
|  | 3 | 5 | 5 | 5 | 5 | 3 |
|  | 2 | 3 | 3 | 2 | 1 | 2 |
|  | 3 | 4 | 4 | 2 | 0 | 2 |
|  | 4 | 5 | 5 | 5 | 2 | 2 |
|  | 3 | 4 | 4 | 4 | 2 | 2 |
|  | 4 | 5 | 5 | 5 | 5 | 3 |
|  | 2 | 3 | 3 | 3 | 2 | 2 |
|  | 3 | 4 | 4 | 2 | 2 | 2 |
|  | 1 | 2 | 2 | 1 | 0 | 1 |
|  | 1 | 2 | 2 | 2 | 1 | 1 |
| 23 °C (n=8) | 2 | 3 | 2 | 0 | 0 | 2 |
|  | 1 | 2 | 1 | 0 | 0 | 1 |
|  | 3 | 4 | 4 | 2 | 2 | 2 |
|  | 1 | 2 | 2 | 2 | 2 | 1 |
|  | 1 | 3 | 3 | 0 | 0 | 2 |
|  | 2 | 3 | 3 | 2 | 0 | 2 |
|  | 4 | 5 | 5 | 5 | 3 | 3 |
|  | 3 | 4 | 4 | 2 | 0 | 2 |
| 28 °C (n=8) | 2 | 3 | 2 | 1 | 0 | 2 |
|  | 0 | 0 | 0 | 0 | 0 | 1 |
|  | 2 | 3 | 4 | 3 | 2 | 2 |
|  | 1 | 2 | 1 | 0 | 0 | 1 |
|  | 0 | 0 | 0 | 0 | 0 | 1 |
|  | 1 | 2 | 2 | 1 | 0 | 1 |
|  | 1 | 2 | 2 | 2 | 2 | 1 |
|  | 3 | 4 | 4 | 1 | 0 | 2 |
| 33 °C (n=4) | 0 | 0 | 0 | 0 | 0 | 1 |
|  | 0 | 2 | 2 | 0 | 0 | 1 |
|  | 0 | 0 | 0 | 0 | 0 | 1 |
|  | 0 | 0 | 0 | 0 | 0 | 1 |

**Table S2.** HE and TUNEL results

| Types | Motor score during reperfusion period | | | | | Neuron number | Tunel-positive score |
| --- | --- | --- | --- | --- | --- | --- | --- |
|  | 1h | 4h | 12h | 24h | 48h |  |  |
| Type 1 | 1 | 2 | 2 | 2 | 1 | 38 | 1 |
|  | 1 | 2 | 2 | 1 | 0 | 18 | 2 |
|  | 1 | 2 | 1 | 0 | 0 | 5 | 2 |
|  | 1 | 2 | 2 | 2 | 2 | 28 | 3 |
|  | 0 | 0 | 0 | 0 | 0 | 24 | 1 |
| Type 2 | 3 | 4 | 4 | 2 | 0 | 6 | 4 |
|  | 4 | 5 | 5 | 5 | 2 | 13 | 3 |
|  | 3 | 4 | 4 | 4 | 2 | 18 | 3 |
|  | 2 | 3 | 3 | 3 | 2 | 16 | 3 |
|  | 3 | 4 | 4 | 2 | 2 | 41 | 3 |
| Type 3 | 4 | 5 | 5 | 5 | 5 | 82 | 1 |
|  | 4 | 5 | 5 | 5 | 3 | 55 | 2 |
|  | 4 | 4 | 4 | 4 | 4 | 55 | 1 |
|  | 4 | 5 | 5 | 5 | 5 | 45 | 1 |
|  | 3 | 5 | 5 | 5 | 5 | 39 | 1 |
